# Calcium Signaling in Macrophages During a Wound Response In Vivo

**DOI:** 10.3390/ijms27104463

**Published:** 2026-05-16

**Authors:** Jordan A. Munos, Pui-Ying Lam

**Affiliations:** Department of Cell Biology, Neurobiology and Anatomy, Medical College of Wisconsin, Milwaukee, WI 53226, USA

**Keywords:** zebrafish, Danionella, macrophages, Calcium

## Abstract

Macrophages are among the earliest responders to tissue injury and remain associated with the wound throughout the healing process. Calcium (Ca^2+^) signaling regulates many immune cell behaviors, yet its role in macrophage responses to injury in vivo remains poorly defined. Here, we used transgenic zebrafish (*Danio rerio*) and *Danionella cerebrum* lines that specifically express the genetically encoded Ca^2+^ indicator, GCaMP, in macrophages. Live confocal imaging was used to monitor macrophage Ca^2+^ dynamics during the early wound response. We found that injury triggers macrophage recruitment to the wound site, where cells exhibit robust and repetitive intracellular Ca^2+^ transients that persist for several hours. Pharmacological perturbation revealed that endoplasmic reticulum Ca^2+^ stores contribute to sustaining these transients, while additional Ca^2+^ sources likely participate in macrophage Ca^2+^ signaling in vivo. Functionally, these Ca^2+^ transients do not appear to be required for chemotaxis, phagocytosis, or TNFα activation during the early stages of wound healing. Together, these findings uncover a previously uncharacterized macrophage Ca^2+^ signaling behavior and highlight the complexity of Ca^2+^ regulation during tissue injury responses in vivo.

## 1. Introduction

Following tissue injury, macrophages rapidly migrate to the wound site, where they initiate key early responses, including phagocytosis and cytokine or chemokine release. Calcium (Ca^2+^) is a ubiquitous second messenger involved in many cellular processes, and is tightly regulated to enable rapid and precise signaling [1]. Macrophage Ca^2+^ signaling have been linked to a wide range of macrophage behaviors, including damage detection [2,3,4,5], activation [6,7], migration [3,8], phagocytosis [9,10,11,12,13], and disease progression [5,14]. Among the diverse forms of Ca^2+^ signaling, Ca^2+^ transients—short, repeated increases in cytosolic Ca^2+^—are of particular interest when displayed by immune cells. These transients can occur across the whole cell or be restricted to specific subcellular regions and typically last from a few seconds to minutes, occurring at frequencies of approximately 0.1–0.5 transients per minute [3,5,10,13]. A wide range of Ca^2+^ transient duration, frequency, and spatial patterns is observed in vitro [8,9,13,15]. However, the in vivo properties of Ca^2+^ transients in macrophages, as well as their functional roles, remain poorly defined. For example, macrophage activation to a pro-inflammatory state has been associated with Ca^2+^-dependent pathways but in vivo imaging studies have largely focused on Ca^2+^ signals within damaged tissue or other immune cell types [2,15,16,17], leaving macrophage-specific signaling events unresolved. Similarly, the involvement of Ca^2+^ in phagocytosis remains debated. Some studies report brief Ca^2+^ transients preceding engulfment [11], whereas others describe whole-cell or subcellular Ca^2+^ events, ranging from transient to sustained signals, occurring during phagocytic cup formation [10,12,18]. This broad functional repertoire raises important questions about how Ca^2+^ transients—often similar in appearance yet variable in duration, frequency, and spatial scale—encode distinct cellular outcomes. Understanding which forms of Ca^2+^ signaling correspond to specific macrophage behaviors and their functional roles in vivo remains a significant gap in the field.

Zebrafish (ZF) and *Danionella cerebrum* (DC) were used in this study to investigate macrophage Ca^2+^ signaling during an in vivo wound response. ZF are a well-established model system, while DC is a closely related and recently adopted vertebrate model for immune studies [19], first introduced for biomedical research in 2018 [20,21]. Both species are optically transparent at larval stages, making them particularly well-suited for live imaging of immune cell dynamics. In addition, zebrafish and DC are amenable to Tol2-mediated transgenesis [22], enabling tissue-specific expression of fluorescent markers and biosensors. Here, we used the macrophage-specific *mpeg1* promoter [23] to drive expression of the genetically encoded Ca^2+^ indicator GCaMP6s [24], which allowed for the visualization of macrophage Ca^2+^ activity in vivo using confocal time-lapse imaging. Tissue injury was induced by tail-fin transection, a well-characterized model for studying immune cell recruitment and behavior over time [25,26,27]. Using this injury model, we observed that robust macrophage Ca^2+^ transients occurred within 50 μm of the wound edge and persisted for several hours after injury. Pharmacological inhibition of Ca^2+^ channels led to a reduction in transient frequency, suggesting that endoplasmic reticulum Ca^2+^ stores contribute to the maintenance of these signals. Functionally, however, there is no clear association between these Ca^2+^ transients and macrophage chemotaxis or phagocytosis, and reducing transient frequency did not impair TNFα induction. The persistence of Ca^2+^ activity despite combinatorial channel inhibition, together with the absence of effects on these functional outcomes, suggests that Ca^2+^ transients from different sources are occurring in the same cell during a wound response and the possible existence of functional redundancies.

## 2. Results

### 2.1. Macrophages Exhibit Increases in Intracellular Ca^2+^ Transients in Response to Injury

**Figure 1:**
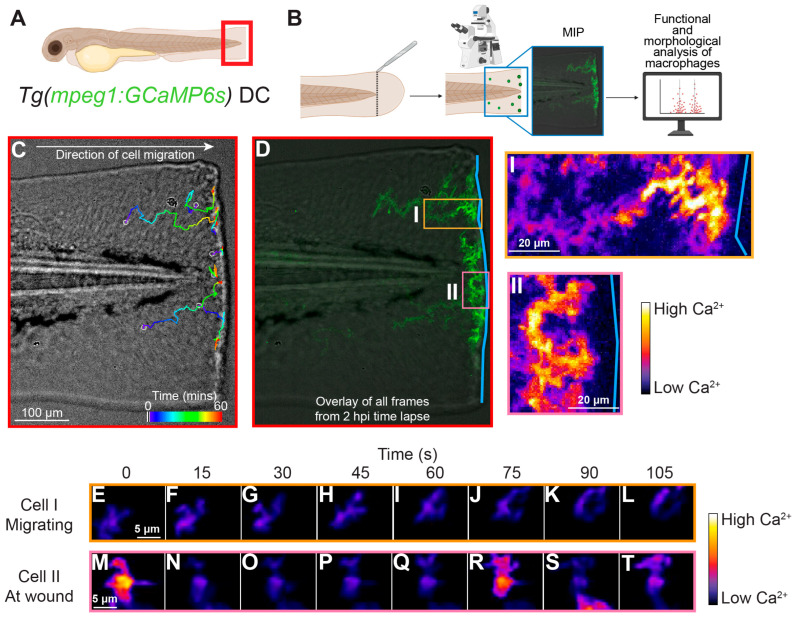
**Macrophages generate Ca^2+^ transients upon tail fin injury.** (**A**) Schematic of the field of view (red box) and transgenic line used to generate the images in (**C**–**T**). (**B**) Schematic overview of the experimental workflow, including injury, imaging, and analysis. MIP, maximum intensity projection. (**C**) Injury-induced macrophage migration to the injury site. Time color-coded tracks of selected macrophages from 2–3 h post injury (hpi) in a one-hour confocal time-lapse movie. Color key: blue indicates the start of the imaging window, and red indicates the end. (**D**) Ca^2+^ response in macrophages over a 1 h time lapse. Ca^2+^ signals increase as cells migrate closer to the wound site. The blue line indicates the wound edge. The orange and pink boxes in D correlate to outsets I and II, as well as individual cells in (**E**–**T**). Outsets (I and II) are pseudocolored for the intensity of the GCaMP6s signal. Color key: white indicates a high GCaMP6s signal and blue indicates a low GCaMP6s signal. Outsets are specific cells from D that illustrate the difference in Ca^2+^ levels between (I) a migrating cell and (II) cells at the wound edge. (**E**–**T**) Time-lapse MIP images illustrating Ca^2+^ levels when a cell is migrating to the wound site (**E**–**L**) compared to when a cell is at the wound site (**M**–**T**) at the time point indicated.

**Figure 2:**
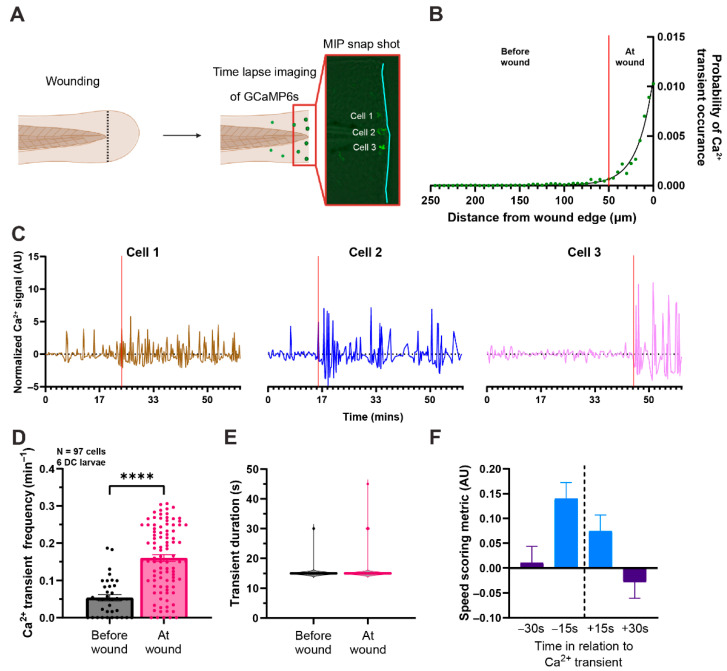
**Macrophages have increased Ca^2+^ transient frequency when cells are within 50 μm from the wound edge.** (**A**) Schematic of the experiment: confocal imaging was performed for 2 h after tail transection. (**B**) Macrophages exhibit most Ca^2+^ transients when within 50 μm from the wound edge. The vertical red line denotes 50 µm from the wound edge. (**C**) Representative GCaMP6s signal traces over time for 3 cells that reach the wound within the 1h time lapse. The vertical red line denotes when the cell is within 50 μm of the wound edge. (**D**) Quantification of the frequency of transients before cells reach the wound and when cells are at the wound site (within 50 µm of the wound edge). Error bars are SEM. (Unpaired *t*-test, *p*-value **** < 0.0001) (**E**) Quantification of Ca^2+^ transient duration. (**F**) Quantification of the macrophage migration speed before and after a transient has occurred at the time point indicated (dashed vertical black line). N = 96 cells from 6 DC larvae for all quantification in (**D**–**F**).

To visualize intracellular Ca^2+^ dynamics in macrophages, we generated a transgenic DC line, *Tg(mpeg1:GCaMP6s)*, expressing the genetically encoded Ca^2+^ reporter GCaMP6s specifically in macrophages. Injury was induced using a tail fin transection assay (Figure 1A,B). Macrophages were imaged using a spinning disk confocal microscope from 0–2 h post injury (hpi). Immediately after injury, macrophages migrated towards the wound, reaching the injury site 15 min after injury (Figure 1C). Migrating cells that were far from the wound displayed only a mild increase in intracellular Ca^2+^ (Figure 1D–L, Supplementary Movie S1). Conversely, macrophages that migrated close to the wound site exhibited repeated increases in intracellular Ca^2+^ over the 2 h imaging window (Figure 1D,M–T, Supplementary Movie S1). Ca^2+^ transients continue beyond the initial 2 hpi period and were observed at 6, 12, and 24 h post injury. Transients were defined as any Ca^2+^ event that exceeded the baseline GCaMP6s signal by at least two standard deviations. Using this definition, we quantified transient occurrence at varying distances from the wound edge. We observed a sharp increase in Ca^2+^ transients when macrophages were approximately 50 μm from the wound edge (Figure 2A,B), indicating a strong relationship between Ca^2+^ activity and macrophage proximity to the wound. Representative Ca^2+^ traces (Figure 2C) showed a similar trend, with Ca^2+^ signals increasing as macrophages approached within 50 μm of the wound (red vertical line) compared to signals when further out. Overall, cells located more than 50 μm from the wound edge exhibited significantly fewer Ca^2+^ transients than cells within 50 μm (Figure 2D). There was no significant difference in the duration of Ca^2+^ transients, regardless of their proximity to the wound (Figure 2E). Further analysis revealed that Ca^2+^ transients were associated with a subsequent decrease in cell migration speed (Figure 2F). Together, these observations suggest that macrophage Ca^2+^ transients are closely linked to their proximity to the wound and correlate with a reduction in migratory velocity.

### 2.2. ER Ca^2+^ Contributes to Macrophage Ca^2+^ Transients

In macrophages, intracellular Ca^2+^ signals arise from two principal sources: extracellular Ca^2+^ influx and release from endoplasmic reticulum (ER) stores [1]. Extracellular Ca^2+^ can enter the cell through plasma membrane pathways involving ATP-dependent P2 receptors or through channels that facilitate store-operated calcium entry (SOCE), such as ORAI1 and TRPC1. Ca^2+^ can also be released from internal sources such as the ER, which is mediated by IP_3_ receptors. Depletion of ER Ca^2+^ is sensed by STIM1, which subsequently activates store-operated Ca^2+^ entry through plasma-membrane channels. In parallel, inhibition of P2Y receptors can reduce IP_3_ production, thereby decreasing ER Ca^2+^ release. Because these channels and receptors have been implicated in macrophage migration [8,15] and activation [3,4,5], we next examined how perturbing specific Ca^2+^ pathways affects the Ca^2+^ transients observed during wound responses.

**Figure 3:**
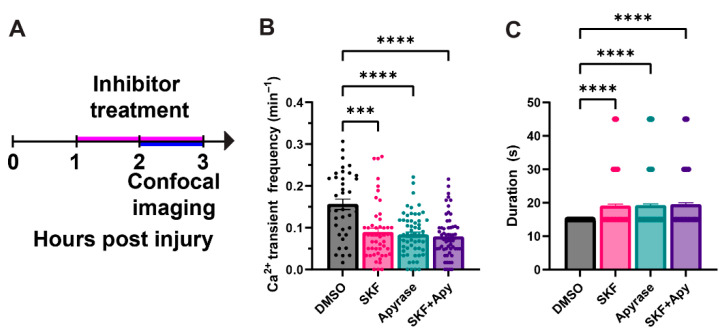
**The endoplasmic reticulum (ER) contributes Ca^2+^ to sustain the macrophage Ca^2+^ transients.** (**A**) Timeline of inhibitor treatment and confocal imaging. (**B**) Quantification of Ca^2+^ transients after different treatment conditions. There is a significant decrease in transient frequency when fish were treated with SKF-96365 (SOCE and TRPC inhibitor) and Apyrase (an enzyme that degrades extracellular ATP) compared to DMSO controls. Error bars are SEM. (**C**) Quantification of Ca^2+^ transient duration across different treatments. (one-way ANOVA, *p*-value *** < 0.0002; **** < 0.0001) N = 39–61 cells from 3–6 DC larvae for quantification in (**B**,**C**). SKF, SKF-96365; Apy, Apyrase.

We reduced ATP-dependent P2 receptor signaling using apyrase, which degrades extracellular ATP, and inhibited store-operated Ca^2+^ entry using SKF-96365, which blocks STIM1-dependent activation of TRPC1/ORAI channels. These approaches allowed us to assess the contribution of extracellular and ER-coupled Ca^2+^ pathways to macrophage Ca^2+^ transients during the wound response. Larval fish were treated with inhibitors at 1 hpi to allow macrophages to reach the wound site before Ca^2+^ channel blockade. Following a 1 h incubation with inhibitors, fish were imaged using time-lapse confocal microscopy for 1 h from 2–3 hpi (Figure 3A).

Both apyrase and SKF-96365 individually reduced the frequency of macrophage Ca^2+^ transients compared with DMSO controls. However, co-treatment with both inhibitors did not result in an additive reduction of transients (Figure 3B). Although the inhibitors act at distinct steps within the Ca^2+^ signaling cascade, the lack of additivity suggests that ATP-dependent P2 receptor signaling and STIM1-dependent Ca^2+^ entry function within a shared, ER-associated pathway required to sustain these transients. Together, these results indicate that ER-derived Ca^2+^ contributes to at least a subset of macrophage Ca^2+^ transients observed during wound responses.

### 2.3. Injury-Induced Ca^2+^ Transients in Macrophages Are Similar in Both Danionella cerebrum (DC) and Zebrafish (ZF)

**Figure 4:**
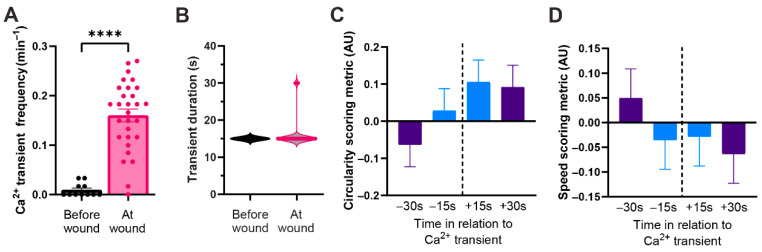
**The generation of Ca^2+^ transients by macrophage after injury is conserved in both DC and ZF.** (**A**) Quantification of macrophage Ca^2+^ transient frequency in ZF. Error bars are SEM. (**B**) Quantification of Ca^2+^ transient duration. (**C**) Quantification of the circularity of macrophage before and after a transient was observed. Error bars are SEM. (**D**) Quantification of the speed of macrophages before and after a Ca^2+^ transient was observed. Error bars are SEM. (Unpaired *t*-test, *p*-value **** < 0.0001) N = 29 cells from 4zf larvae for all quantification in (**A**–**D**).

To determine whether wound-induced macrophage Ca^2+^ transients are conserved across other vertebrate species, we performed the same analyses using the transgenic ZF line *Tg(mpeg1:Gal4, UAS:GCaMP5)*. Similar to observations in DC macrophages, ZF macrophages displayed Ca^2+^ transients when they migrated to within 50 μm of the wound edge (Figure 4A). The average transient duration was also comparable, with average events lasting approximately 15 s (Figure 4B). The zebrafish transgenic line used for these experiments exhibits sparse macrophage labeling, allowing us to characterize the relationship between cell morphology, migration speed, and Ca^2+^ transients. This analysis was more challenging in DC due to dense macrophage clustering post injury. Using this zebrafish line, we quantified cell circularity and migration speed before and after each transient. A scoring system was developed to correlate cell circularity and migration speed with the generation of Ca^2+^ transients. Scores ranged from +1 to −1: +1 indicated that cells were rounder or faster, −1 indicated that cells were less round or slower, and 0 indicated no change. Scores were calculated at 30 s and 15 s before and after each transient to assess temporal dynamics. We found that cells became rounder and moved more slowly following a transient compared to before (Figure 4C,D). In sum, cell shape and speed showed an inverse relationship with Ca^2+^ transient generation. That is, cells were less round and faster beforehand, and rounder and slower afterward. Similar results from both ZF and DC suggests that macrophages exhibit conserved in vivo Ca^2+^ transient responses to injury.

### 2.4. Early Macrophage Activation Is Unaffected by Inhibition of Ca^2+^ Transients

**Figure 5:**
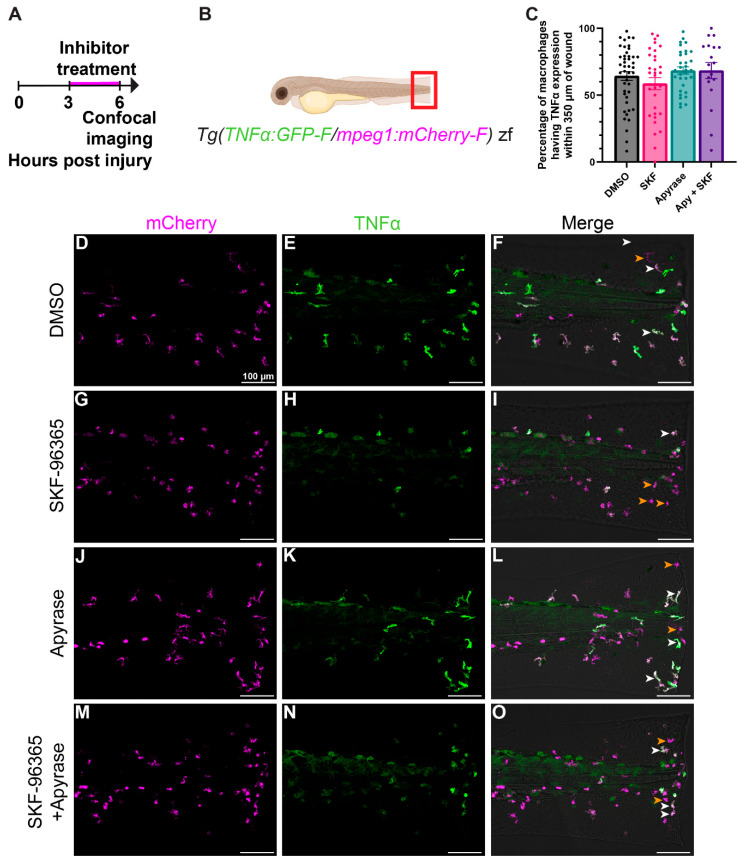
**Decreasing Ca^2+^ transients does not affect TNFα expression in macrophages.** (**A**) Timeline of the experiment. (**B**) Schematic showing the field of view (red box) imaged in (**D**–**O**). (**C**) Quantification of the percentage of TNFα-expressing macrophages within 350 μm of the wound edge at 6 hpi with different treatments. There was no statistical significance between treatment groups, suggesting the Ca^2+^ transients inhibited by treatment are not involved in the upregulation of TNFα expression in macrophages after injury. Error bars are SEM. N = 18–45 larvae. (**D**–**O**) Representative maximum intensity projection confocal images of the different treatment groups. White arrowheads indicate cells expressing both mCherry and GFP. Orange arrowheads indicate cells only expressing mCherry.

Ca^2+^ signaling has been linked to macrophage activation during the early stages of an immune challenge. However, most supporting evidence comes from studies using electrophysiology on cultured cells, therefore, in vivo Ca^2+^ dynamics remain unclear [6,7]. In vitro, increased TNFα expression—a hallmark of early macrophage activation—has been shown to depend on transient increases in intracellular Ca^2+^ [28,29]. We therefore asked if the Ca^2+^ transients observed after injury contribute to early TNFα expression in macrophages using a transgenic TNFα reporter as a proxy for activated macrophages. To visualize TNFα activity, we used *Tg(TNFα:GFP-F, mpeg1:mCherry-F)*, transgenic zebrafish, where GFP-F reports TNFα promoter activity and mCherry-F labels macrophage cell bodies (Figure 5B) [30]. After tail-fin transection, fish were maintained under standard conditions to allow macrophage recruitment for 3 h. Fish were then exposed to DMSO or the same Ca^2+^ channel inhibitors previously described (see Figure 3B) from 3–6 hpi before being imaged (Figure 5A,B). We manually quantified the total number of mCherry^+^ and GFP^+^/mCherry^+^ macrophages that were within 350 μm of the wound edge. The percentage of GFP^+^/mCherry^+^ cells was then calculated as a measure of TNFα activation. We found that the proportion of TNFα-expressing macrophages remained unchanged across all inhibitor treatments compared to DMSO control (Figure 5C–O), indicating that the Ca^2+^ transients sensitive to these compounds are not required for early macrophage activation during the wound response.

### 2.5. Phagocytic Engulfment Occurs Largely Without Detectable Ca^2+^ Transients

**Figure 6:**
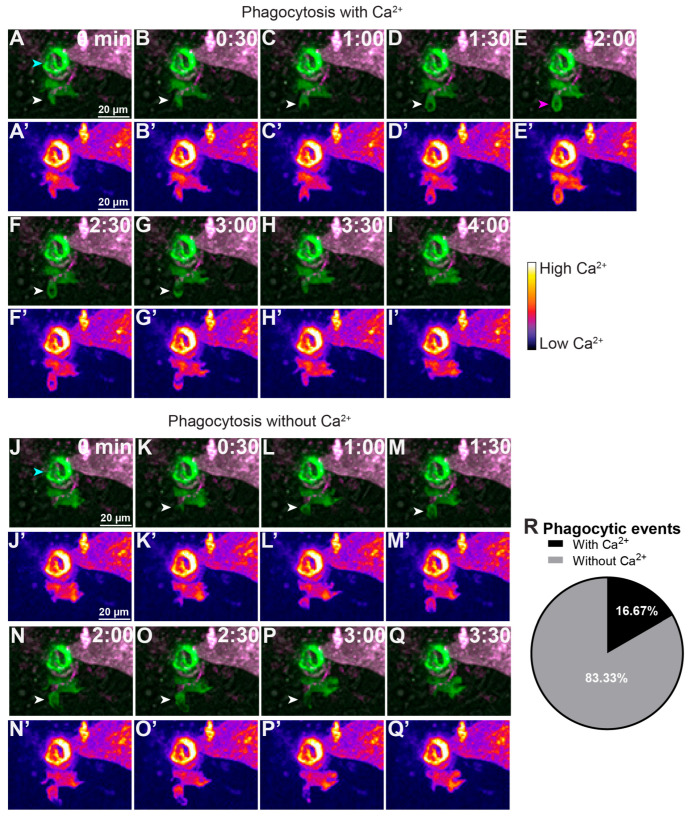
**Macrophage Ca^2+^ imaging during phagocytic events in vivo**. Confocal time lapse imaging was performed post laser-induced injury in *Tg(mpeg1:GCaMP6s)* DC larvae with transient mosaic expression of tdTomato in keratinocytes. (**A**–**Q**) MIP images at the time point indicated illustrating phagocytic events that occur with a Ca^2+^ transient event (**A**–**I**) or without a Ca^2+^ transient event (**J**–**Q**). White arrowheads indicate the formation of a phagocytic cup. Pink arrowhead in E indicates when a calcium transient occurs. Blue arrowheads in (**A**,**J**) indicate the location of laser injury. (**A**’–**Q**’) The same time lapse images from (**A**–**Q**), but color-coded for the level of Ca^2+^ in the cell during the specified time points. (**R**) Percentage of phagocytic events observed and the correlation to the occurrence of macrophage Ca^2+^ transients. N = 20 phagocytic events imaged from eight larvae.

Multiple stages of phagocytosis have been associated with Ca^2+^ signaling both in vitro and in vivo. In vitro studies have linked Ca^2+^ to the initiation [11] and efficiency [9] of phagocytosis, while in vivo work has shown that Ca^2+^ is involved in the engulfment phase in macrophages [10]. To investigate if the Ca^2+^ transients we observe are required for phagocytosis, we injected *Tg(mpeg1:GCaMP6s)* DC embryos with *krt4:tdTomato*. The *krt4* promoter [31] drives tdTomato expression in superficial epidermal cells. To limit macrophage recruitment and facilitate clearer analysis of phagocytosis, we performed laser ablation of individual tdTomato^+^ cells. Fish were imaged immediately after injury for 3 h. Phagocytic events were manually scored by counting phagocytic cup formation and closure in the presence or absence of Ca^2+^ transients. We observed that while phagocytic cups can form and close during Ca^2+^ transients (Figure 6A–I,R), most phagocytic events occurred without any detectable Ca^2+^ transient (Figure 6J–R). These findings indicate that the Ca^2+^ transients described here are not required for phagocytosis.

## 3. Discussion

Macrophage recruitment to sites of injury is an early and essential step in wound healing. Ca^2+^ is an important second messenger involved in many macrophage functions. Key macrophage functions during the early stages of wound response include damage sensing, migration, activation, and phagocytosis of cellular debris, all of which have been linked to Ca^2+^ signaling. Despite this importance, intracellular Ca^2+^ dynamics in immune cells remain poorly characterized in vivo. It has been recorded that damaged tissue in both mice and zebrafish exhibits a wave of Ca^2+^ immediately following damage [2,16,17]. However, the properties and functional significance of injury-induced Ca^2+^ transients in macrophages in vivo have remained largely unexplored. One notable example reported whole-cell Ca^2+^ flux in neutrophils when they approached within ~50 μm of a wound site, though the Ca^2+^ source and functional relevance of these signals were unclear [18]. In this study, we systematically characterized macrophage Ca^2+^ dynamics during a wound response and identified Ca^2+^ transients that occur when macrophages are within approximately 50 µm of the wound edge. Pharmacological inhibition using apyrase, which degrades extracellular ATP, reduced the frequency of these transients, suggesting that ATP released from damaged tissue contributes to their initiation. Additional pharmacological perturbations further pointed to ER Ca^2+^ stores as a major source sustaining these intracellular Ca^2+^ transients.

To elucidate the functional role of these signals, we examined whether macrophage Ca^2+^ transients are involved in chemotaxis, activation, or phagocytosis. With regard to chemotactic migration, a Ca^2+^ gradient has been shown to support directional migration in neutrophils in vivo [18]. However, we observed that macrophage Ca^2+^ transients occurred only rarely during directed migration toward the wound site. This contrasts with a report by Du et al., (2022) in microglia, the resident macrophages of the brain, where Ca^2+^ bursts accompany migration in response to focal neuronal damage [3]. Notably, Du et al., (2022) relied on a similar genetically encoded Ca^2+^ indicator, and in our work, we observed comparable Ca^2+^ transients using both GCaMP5 and GCaMP6s [3]. The discrepancy is therefore unlikely to be explained by reporter sensitivity and instead may reflect differences between the signaling mechanisms present in peripheral macrophages versus microglia, which operate in distinct tissue environments. Because the Ca^2+^ transients described here represent whole-cell Ca^2+^ elevations and are infrequent during chemotactic movement, we conclude that they are unlikely to be required for macrophage migration toward the wound.

We next examined the role of Ca^2+^ transients in macrophage activation. TNFα is a well-established marker of early macrophage activation and polarization during a wound response [30,32]. In vitro studies have demonstrated that macrophage polarization states depend on Ca^2+^ entry through distinct channels, with pro-inflammatory activation linked to TRPC1-mediated Ca^2+^ flux and anti-inflammatory activation associated with ORAI1-dependent pathways [6,7]. However, these studies did not directly visualize intracellular Ca^2+^ dynamics. In vivo imaging studies have also shown that injury triggers a Ca^2+^ wave that propagates through damaged tissue and is thought to activate macrophages and microglia [2,16,17]. Our results indicate that the Ca^2+^ transients observed within macrophages, in particular those that can be reduced pharmacologically, are not required for early TNFα induction. This suggests that macrophage activation may instead rely on tissue-level Ca^2+^ signals or on sustained, low-level changes in intracellular Ca^2+^ that are distinct from the transient events described here. Given the inability to completely abolish Ca^2+^ transients using pharmacological interventions, together with the absence of a detectable role in the macrophage functions examined here, we hypothesize that both intracellular and extracellular calcium sources act cooperatively to sustain the observed Ca^2+^ transients. In addition, functional redundancy among calcium-dependent signaling pathways may contribute to the partial suppression of calcium activity and the lack of overt functional phenotypes. Future studies employing source-specific calcium biosensors or dyes in combination with cytosolic GCaMP will enable direct testing of whether multiple Ca^2+^ sources jointly maintain these transients. Furthermore, simultaneous perturbation of multiple pathways converging on shared functional outcomes will be essential to rigorously evaluate the contribution of signaling redundancy.

Phagocytosis is another macrophage function frequently associated with Ca^2+^ signaling, though its precise role remains controversial. Recent work in adult zebrafish demonstrated that macrophage Ca^2+^ responses during phagocytosis are size-dependent, with small debris inducing brief Ca^2+^ signals on a time scale of a few minutes and engulfment of large cells producing prolonged Ca^2+^ elevations lasting up to an hour [10]. In our injury paradigm, we detected no consistent association between phagocytic cup formation or closure, and Ca^2+^ transients. If size-dependent Ca^2+^ signaling applies here, interactions with small debris may elicit Ca^2+^ signals below the temporal or spatial resolution of our imaging. Nevertheless, for the phagocytic events we were able to detect, Ca^2+^ transients were not obligate. Notably, studies in neutrophils have reported short Ca^2+^ transients during bacterial phagocytosis, with durations lasting 20–30 s, similar to those observed here [18]. Given that our injury model is not in a sterile environment, some Ca^2+^ transients could reflect macrophage interactions with bacteria or other immunogens. Testing this possibility will require future experiments using fluorescently labeled pathogens. Additionally, in vitro studies using IgG-coated beads have shown Ca^2+^ transients immediately preceding phagocytosis, with one study [11] suggesting that Ca^2+^ signaling requirements may vary depending on the phagocytic trigger.

Although the Ca^2+^ transients observed in this study did not appear to directly drive chemotaxis, activation, or phagocytosis, we observed a trend toward decreased migration speed and increased cell roundness following the onset of Ca^2+^ transient generations (Figure 4C,D). While these changes did not reach statistical significance, they are consistent with a role for Ca^2+^ in regulating cytoskeletal dynamics. Transient Ca^2+^ signals are known to drive cytoskeletal reorganization and changes in cell polarity in motile cells [33,34], though little work has addressed these processes in macrophages in vivo. It is therefore plausible that Ca^2+^ transients contribute to transient pauses or polarity changes as macrophages interact with the wound environment and fine-tune their response.

Although chemotaxis, phagocytosis, and cellular activation are key macrophage responses during wound healing, they represent a subset of functions involved. Investigating additional macrophage activities, such as the detection of danger-associated molecular patterns (DAMPs), detecting and responding to chemoattractants, and interacting with other cell types, could further clarify the role of these Ca^2+^ transients. The use of pharmacological inhibitors or genetically encoded biosensors combined with GCaMP would enable more precise investigation of macrophage functions not examined here but are known to be critical to the wound response. For example, GCaMP-facilitated macrophage Ca^2+^ imaging can be combined with the Leukotriene B_4_ (LTB_4_) biosensor GEM-LTB_4_ [35] to determine the involvement of Ca^2+^ transients in chemoattraction. Fluorescent labeling of other cell types at the site of injury, such as neutrophils with the *lysC* promoter [36], could help determine if transients facilitate cell-cell interaction.

It should be considered that the observed Ca^2+^ transients may represent a form of macrophage “pre-activation” or priming state that sensitizes cells to subsequent inflammatory or injury cues, rather than driving immediate effector functions. Additionally, emerging literature suggests that innate immune cells, including macrophages, can exhibit forms of immune memory or trained immunity [37,38]. Therefore, persistent calcium signaling may contribute to longer-term changes in responsiveness or phenotypic bias that manifest only upon secondary challenge. To test this theory, future investigations could include combining small-molecule inhibition of Ca^2+^ transients with secondary injury or infection.

In summary, we used in vivo time-lapse imaging to identify a previously unreported form of macrophage Ca^2+^ signaling during a wound response. Macrophages display Ca^2+^ transients that occur when they are within proximity (~50 μm) of a wound edge and partially depend on ER Ca^2+^ stores. These transients inhibited with small molecules seem dispensable for phagocytosis and TNFα activation. Together, our results emphasize the complexity of macrophage Ca^2+^ signaling in vivo and suggest that Ca^2+^ transients with similar phenotypes may originate from multiple Ca^2+^ sources.

This study has several limitations. Our imaging approach measures global cytosolic calcium levels, whereas many macrophage effector functions are known to depend on spatially restricted calcium microdomains, such as those formed near phagosomes or ER–mitochondria contact sites. As such, functionally relevant local calcium signaling may not be resolved in our datasets. In addition, due to imaging constraints, our temporal resolution was limited to one z-stack every 15 s, meaning that faster Ca^2+^ transients or highly localized events may have gone undetected and that average transient duration may be overestimated. Nevertheless, macrophages exhibited normal behavior throughout imaging, indicating minimal phototoxicity. Additionally, pharmacological treatments only partially suppressed Ca^2+^ transients, raising the possibility of redundant Ca^2+^ sources or signaling pathways. Complete inhibition was achievable with a cocktail of inhibitors but resulted in adverse effects on larval health, precluding functional analysis. Moreover, pharmacological inhibitors inherently lack cell specificity and could suppress Ca^2+^ signaling not only in macrophages but also in other, unimaged cell types, complicating the interpretation of downstream effects. Macrophage-specific perturbations in future studies employing macrophage-specific genetic approaches, optogenetics, or photopharmacological tools such as TRPswitch [39,40] to modulate Ca^2+^ signaling will be essential to fully identify the cell specific roles played by these injury-induced Ca^2+^ transients. Finally, although our work focused on larval animals, the generation of DC transgenic lines opens the door to future investigations of macrophage Ca^2+^ dynamics in adult animals due to *D. cerebrum*’s lifelong transparency, where increased tissue complexity may reveal additional layers of regulation.

## 4. Materials and Methods

### 4.1. Animal Husbandry

Adult *Danionella cerebrum* (DC) and zebrafish (*Danio rerio*) were maintained and bred under normal conditions (28.5 °C and 14 h/10 h light/dark cycle) as previously described [19,20,21,41]. Larvae were kept in 10 cm Petri dishes with E3 and 10 mM HEPES (GoldBio (St. Louis, MO, USA), cat no. H-400-500) at 28 °C with a 14 h/10 h light/dark cycle. These conditions are in accordance with protocols approved by the Medical College of Wisconsin Institutional Animal Care and Use Committee (IACUC).

### 4.2. Creation of Transgenic GCaMP6s Expressing DC Line

The *Tol2-mpeg1:GCaMP6s* transgenesis vector was generated using Gateway cloning. Briefly, the LR Clonase II Plus enzyme (ThermoFisher Scientific (Waltham, WA, USA), cat. no. 12538120) was used to combine p5E-mpeg1 [23] (Addgene (Watertown, MA, USA) 75023), pME-GCaMP6s [24], p3E-polyA [22], and pDestTol2CG [22]. Transgenesis was achieved by injecting 2–3 nL of a solution containing 9 ng/µL of construct DNA and 12.6 ng/µL in vitro transcribed (Ambion (Waltham, WA, USA)) Tol2 transposase mRNA into DC embryos at the 1–4 cell stage. F0 injected larvae showing strong transient expression were grown to adulthood and used to establish a stable transgenic line.

### 4.3. Chemicals

Dimethyl sulfoxide (DMSO) was purchased from Fisher Scientific (Pittsburgh, PA, USA; cat. no. BP231-100) and used as a negative control at 0.01% of the final volume. All compounds were dissolved in DMSO unless otherwise stated, and stock solutions were prepared accordingly. SKF-96365 (cat no. 10009312, 2 mM) was purchased from Cayman Chemical Company (Ann Barbor, MI, USA). Apyrase (A6535, 333 U/mL) dissolved in PBS was purchased from Millipore Sigma (Burlington, MA, USA).

### 4.4. Confocal Live Imaging

Larvae were mounted laterally on a glass bottom plate (Cellvis (Mountain View, CA, USA), cat no. D35-20-0-N) using 1% low melt agarose (LMA; Genesee Scientific (El Cajon, CA, USA), cat no. 20-104 or GoldBio, cat no. A-204-100). Embedded larvae were covered in 2 mL of E3 with tricaine (0.74 mM, Fisher Scientific, cat no. NC0342409) and imaged on a Nikon Ti2 inverted microscope equipped with a Yokogawa CSU-W1 spinning disk confocal with a 20×0.75 NA air objective (Nikon (Shinagawa, Japan)). Samples were kept at ~28.5 °C while imaging using a STXG stage top incubator (Tokai Hit (Bala Cynwyd, PA, USA)). GCaMP signal was captured using a 488 nm excitation laser and a FITC filter (emission 515–555 nm). Z stacks using a 0.9 μm z steps were acquired every 15 s in 1 h intervals for 3 h post-injury (hpi) with the following parameters: 100 ms exposure, 2 × 2 binning, and 16-bit depth.

### 4.5. Tail Transection

For tail fin wound assays, transgenic DC *Tg(mpeg1:GCaMP6s)* or zebrafish *Tg(mpeg1:Gal4, UAS:GCaMP5)* larvae were used. Larval fish were screened for transgene expression at 2 days post-fertilization (dpf) and used in experiments at 3 dpf unless otherwise specified. Larvae were anesthetized in E3 containing 0.74 mM tricaine prior to tail fin transection. The tail fin transection was performed under a dissecting microscope using a surgical scalpel (Millipore Sigma, cat. no. S2646-100EA) with a vertical cut made at the base of the notochord. Immediately after tail injury, larvae were mounted laterally onto a glass bottom plate for imaging as described above.

### 4.6. 2-Photon Injury

*Tg(mpeg1:GCaMP6s)* DC embryos were injected with 2–3 nL of a solution containing 9 ng/μL of *krt4:Tdtomato* DNA plasmid [42] and 12.6 ng/µL in-vitro transcribed (Ambion) Tol2 transposase mRNA at the 1–4 cell stage for transient mosaic expression. Larvae at 3–4 dpf were mounted as described above. A laser ablation injury was induced using a Nikon AXR upright multiphoton system, equipped with AX-NEU non-descanned detector, dual-output tunable laser (680–1300 nm), fixed laser (1040 nm), and APO 25×W MP IR objective optimized for deep tissue multiphoton imaging. Visualization of tdTomato-positive krt4 cells was achieved using a tunable laser at 920 nm. A circular region of interest (ROI) (radius of 2.5–5.10 μm) was drawn and placed near one edge of the cell. To induce an injury, the following conditions were used with the tunable laser: 820 nm at 3% (2.47 W) and the ROI was scanned for 1s, dwell time 2 μs or 850 nm at 10% (2.48 W) and the ROI was scanned for 10 s with a dwell time of 2 μs. After injury, samples were imaged using a spinning disk confocal as described above with the following changes: a 40×/1.15 NA water objective (Nikon). Z stacks using a 0.8–0.9 µm spacing were acquired every 30 s in 1 h intervals for 3 h post injury.

### 4.7. Ca^2+^ Inhibitor Treatment at the Wound Site

Transgenic *Tg(mpeg1:GCaMP6s)* DC larvae at 3 dpf were used for all inhibitor treatment experiments. Tail transections were performed as described above. Immediately after injury, larvae were transferred to a 35 mm Petri dish (GenClone (El Cajon, CA, USA), cat. no. 32–103) containing 2 mL of E3. Dishes were placed in a 28 °C incubator for 1 h, then treated with Ca^2+^ channel inhibitors or DMSO for 1 h at 28 °C. During imaging, larvae remained exposed to the respective treatments. Imaging was performed as described above except that agarose surrounding the tail was removed to facilitate compound exposure.

### 4.8. TNFα Reporter Imaging

*Tg(TNFα:GFP-F/mpeg1:mCherry-F)* ZF [30] were used for these experiment. Larvae were injured using the tail transection method described above. Following injury, larvae were transferred to E3 medium and incubated at 28 °C for 3 h (0–3 h post injury, hpi). After this initial incubation, compounds were added to the E3, mixed thoroughly, and larvae were incubated for an additional 3 h (3–6 hpi). Imaging was performed as described above with the addition of capturing the mCherry signal using a 561 nm excitation laser and a TRITC filter (577–630 nm). Only a single z-stack was acquired per larva, with no time-lapse imaging. Quantification was carried out by manually counting cells expressing mCherry and cells co-expressing mCherry and GFP.

### 4.9. Analysis

Images were acquired using Nikon NIS Elements and processed in Imaris version 10.0. Analysis was performed on maximum intensity projections. In Imaris, the Spots and Surfaces functions were used to identify and track individual cells over time, and to export GCaMP signal intensity, position data, and cell speed. The following functions were used to normalize the calcium signal and to define calcium transients:

Determining calcium transient:

$$Intensity=\;\frac{F-F0}{F0}$$

The GCaMP6s signal (F) for each frame was sorted from lowest to highest, and the average of the lowest 100 frames was calculated to obtain F0, which was considered background fluorescence. The intensity of the cell for each frame was calculated using the equation above, and the standard deviation (SD) of the signal was calculated. Calcium transients were defined as any intensity value ≥ 2 SD above average fluorescence. Only cells that migrated to within 50 µm of the wound edge during the time-lapse acquisition were included in the analysis. An R code was written to automate all calculations.

Normalization of calcium signal:

F−F0=normalized value

The GCaMP6s signal (F) was normalized to the background signal (F0) using the equation above. After the calcium transient was determined and the signal normalized, a custom R code LOESS (Locally estimated scatterplot smoothing) was used to account for signal bleaching over time.

### 4.10. Statistical Analyses

All results are expressed as means ± SEM. Unless otherwise indicated, an unpaired t-test was used to determine *p*-values. The criterion for statistical significance was *p* < 0.05. Statistical analysis was performed using Prism 9.5 (GraphPad).

## Data Availability

Further raw data can be obtained from the authors upon request.

## Supplementary Materials

The following supporting information can be downloaded at: https://www.mdpi.com/article/10.3390/ijms27104463/s1. Supplementary Movie S1. Corresponding to Figure 1. Macrophage Ca^2+^ response to tail transection injury. 1 h time lapse imaging of 3 dpf *Tg(mpeg1:GCaMP6s)* DC larvae at 2–3 hpi.
